# A new CFD based non-invasive method for functional diagnosis of coronary stenosis

**DOI:** 10.1186/s12938-018-0468-6

**Published:** 2018-03-22

**Authors:** Xinzhou Xie, Minwen Zheng, Didi Wen, Yabing Li, Songyun Xie

**Affiliations:** 1 Department of Electronic Science and Technology, Northwestern Polytechnical University, 127 West Youyi Road, Xi’an, Shaanxi People’s Republic of China; 2 Department of Radiology, Xijing Hospital, Fourth Military Medical University, 15 West Changle Road, Xi’an, Shaanxi People’s Republic of China

**Keywords:** Functional coronary stenosis, Non-invasive assessment, Pressure-flow curve, CFD

## Abstract

**Background:**

Accurate functional diagnosis of coronary stenosis is vital for decision making in coronary revascularization. With recent advances in computational fluid dynamics (CFD), fractional flow reserve (FFR) can be derived non-invasively from coronary computed tomography angiography images (FFR_CT_) for functional measurement of stenosis. However, the accuracy of FFR_CT_ is limited due to the approximate modeling approach of maximal hyperemia conditions. To overcome this problem, a new CFD based non-invasive method is proposed.

**Methods:**

Instead of modeling maximal hyperemia condition, a series of boundary conditions are specified and those simulated results are combined to provide a pressure-flow curve for a stenosis. Then, functional diagnosis of stenosis is assessed based on parameters derived from the obtained pressure-flow curve.

**Results:**

The proposed method is applied to both idealized and patient-specific models, and validated with invasive FFR in six patients. Results show that additional hemodynamic information about the flow resistances of a stenosis is provided, which cannot be directly obtained from anatomy information. Parameters derived from the simulated pressure-flow curve show a linear and significant correlations with invasive FFR (r > 0.95, P < 0.05).

**Conclusion:**

The proposed method can assess flow resistances by the pressure-flow curve derived parameters without modeling of maximal hyperemia condition, which is a new promising approach for non-invasive functional assessment of coronary stenosis.

## Background

Functionally significant stenosis generally causes angina symptoms, and is associated with inducible ischemia and impaired outcome. Therefore, it should be revascularized. While, if a stenosis has no functional significance, medical treatment is quite excellent [1]. Therefore, determining the functional significance of a coronary stenosis plays a pivotal role in decision making in coronary revascularization [1]. Fractional flow reserve (FFR) is considered as the gold standard for assessment of functional measurement of stenosis [2, 3]. It is a pressure derived index and defined as the ratio between the distal and proximal pressure of the stenosis at maximal hyperemia [4]. A cut-off value of 0.75–0.8 is employed to indicate the functionally significant stenosis [3]. FFR-guided strategy has been proved to be safety and also has been demonstrated to be both cost-effective and cost-saving [5–7]. However, FFR is an invasive procedure and requires pharmacologic intervention to induce maximal hyperemia, which limits its in-hospital utilization [2, 8]. To overcome this problem, a novel technology that combines coronary computed tomography angiography (cCTA) and computational fluid dynamics (CFD) has been developed [9–11]. By using the CFD method, an accurate reconstruction of coronary flow and pressure fields can be obtained from cCTA images, and then, the coronary diagnostic index (CT-derived computed FFR, FFR_CT_) can be derived without additional medications [9, 12–15]. Multiple clinic trials have demonstrated that the performance of FFR_CT_ was superior to cCTA stenosis for diagnosing ischemic lesions [16–21]. The use of FFR_CT_ is showed to reduce the overall use of invasive angiography and more and more researchers suggest that FFR_CT_ can be the gatekeeper to the cardiac catheterization laboratory [13].

Pressure drop across a stenosis can be approximately determined by a common fluid dynamic equation [22, 23]:

1 $$\Delta \overline{p} = f\overline{Q} + s\overline{Q}^{2}$$

where $\Delta \overline{p}$ is the mean pressure drop, *f* the is the viscous friction, *s* is the expansion loss and $\overline{Q}$ is the mean flow rate. Coronary stenosis will increase the viscous friction and expansion loss of the stenosis section, leading to an increase in pressure drop. The pressure derived index (FFR and FFR_CT_) quantify the functionally significant of stenosis by using the pressure drop. However, the pressure drop relies on the flow rate, which is also determined by the behavior of distal vascular trees (micro-vascular resistance) [2, 23]. Therefore, to exclude the influence of the flow rate, FFR and FFR_CT_ should be measured during maximal hyperemia. Failure to achieve maximal hyperemia would result in an inaccuracy of FFR [1]. Additional, it will be confounded by the presence of microvascular disease [1]. Several FFR based hyperemia-free indices (basal FFR and instantaneous wave-free ratio) have been proposed for detecting coronary stenosis under basal conditions [24, 25]. However, similar to FFR, the flow rate is still an uncontrollable factor which has great impact on these indices. Since the main principle of FFR_CT_ is to model the blood flow in coronary during the hyperemia condition [9], an accurate modeling of the hyperemia condition is critical. Several physiological models are used in the modeling process: to obtain the baseline coronary flow, a fixed relationship between the baseline flow and the left ventricular myocardial mass is assumed; to get the microvascular resistance, a fixed relationship between the resistance in baseline and hyperemia condition is assumed [9]. These physiological models provide a general approach to model the hyperemia flow rate and make it possible to compute FFR_CT_ without any other medications. However, these models reflect the average behaviors in coronary circulation, ignoring the individual difference. Obviously, these assumptions would reduce the reliability in modeling the individual flow rate during hyperemia condition, which further degrades the accuracy of FFR_CT_.

To overcome this problem, a new CFD based non-invasive approach for functional measurement of stenosis is proposed. It is based on an invasive non-dimensional index, pressure drop coefficient (CDP), which combining both the pressure and flow velocity information. CDP is defined as the ratio of trans-stenotic pressure drop to distal dynamic pressure during maximal hyperemia [26]. Compared with pressure-derived indices (FFR), CDP directly quantifies the hemodynamic behavior of a stenosis section [26]. Thus, the presence of microvascular disease has limited impact on CDP [27–29]. However, due to the limitation of the invasive procedure (the viscous loss is not considered in order to get a result with a single measurement; the flow velocity is measured, but not the flow rate), CDP is still dependent on the flow rate across a stenosis [30]. Although previous studies reported that CDP were useful during both basal and hyperemia condition, the cutoff values were quite different in those two conditions [27, 30]. Additional, guide wire insertion has great impact on flow patterns, and reliable measurement of flow velocity is also technically difficult [23]. Fortunately, those limitations of CDP can be overcame by combining with CFD method. The viscous loss can be included with multiple measurements, simply by specifying different boundary conditions; flow rates also can be accurately obtained without the influence of guide wire insertion. Unlike FFR_CT_, the proposed approach do not need to model the hyperemia condition. Instead, the stenosis flow with a series of flow rates will be simulated and the pressure-flow relationships is obtained. Then, *f* and *s* in Eq. 1 can be estimated and further employed to functional measurement of stenosis.

## Methods

### Vascular geometry reconstruction

**Fig. 1:**
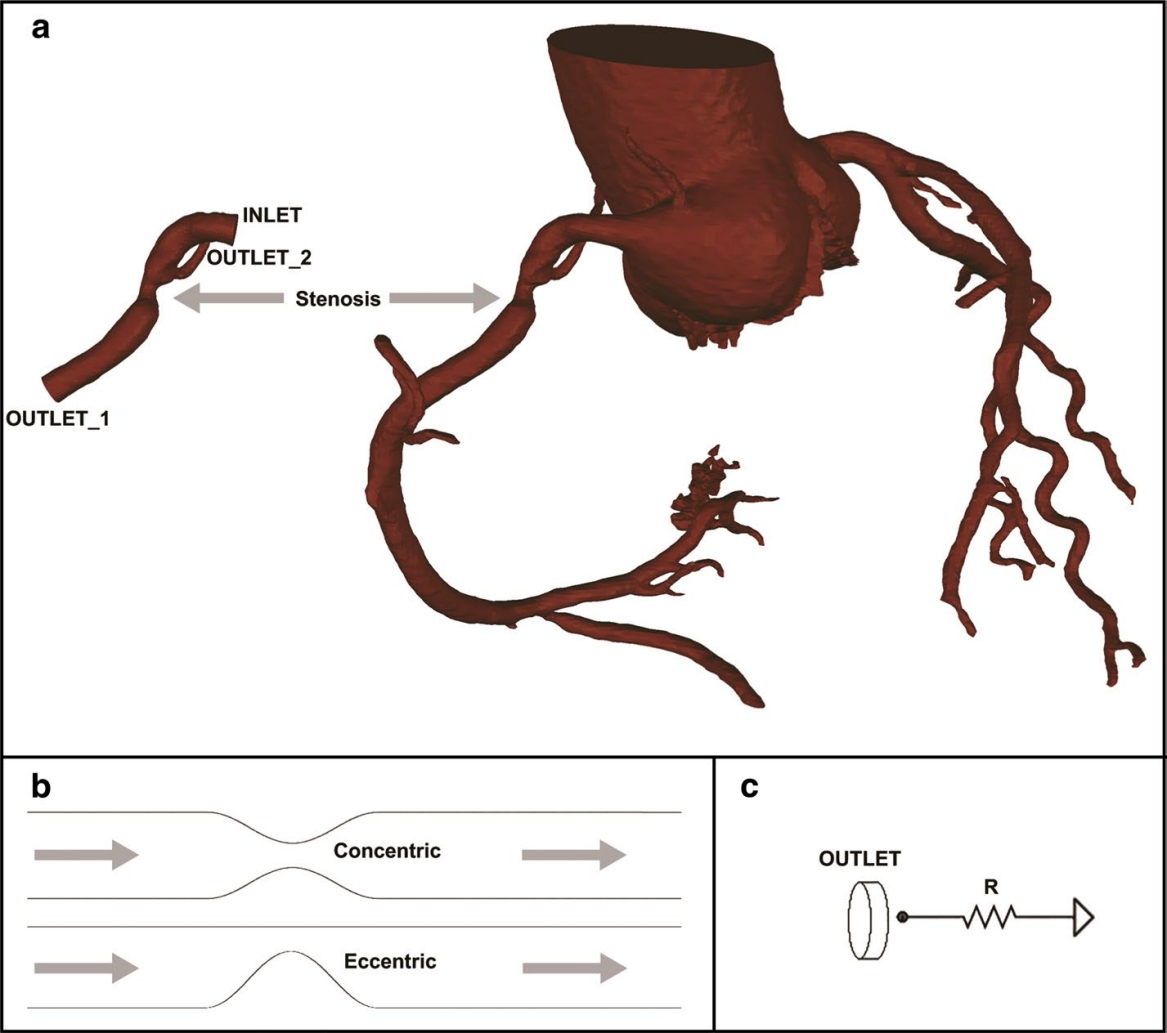
Vascular geometry and boundary conditions. **a** Demonstration of reconstructed patient-specific models (only the stenosis sections with nearby branches are retained for further CFD analysis); **b** demonstration of the two types idealized models (concentric shaped stenosis and eccentric shaped stenosis); **c** outlet boundary condition (a lumped parameter model with only one resistance is coupled to each outlet)

In total, 20 3D coronary models with stenosis (one right coronary artery (RCA), one left circumflex coronary artery (LCX) and 18 left anterior descending coronary arteries (LAD)) have been reconstructed from the cCTA images of 19 patients. The cCTA images are acquired in mid-diastole (60–70% of R–R interval) or systole (40–50% of R–R interval) with 0.4-mm slice interval and 0.28 mm/pixel in-plane image resolution, using a second-generation DSCT (SOMATOM Definition Flash, Siemens Healthcare, Forchheim, Germany) scanner. Coronary arteries are semi-automatically segmented and reconstructed in Mimics (Mimics, Materialise, Leuven, Belgium). And then, only the stenosis sections with nearby branches are retained for further CFD analysis (as shown in Fig. 1a). Area stenosis (AS%) and minimal lumen area (MLA) are calculated based on the reconstructed coronary artery model. AS% is defined as 100% minus the percentage of minimal lumen area to the reference lumen area. The reference site is in close proximity to the lesion, without intervening branch vessels.

Besides the reconstructed patient-specific models, several idealized coronary stenosis models are also created. As shown in Fig. 1b, two types of stenosis are modeled: concentric shaped stenosis and eccentric shaped stenosis. For each types, seven models with the AS% of 65, 70, 75, 80, 85, 90 and 95% are created. These idealized stenosis sections are modeled as 3D pipes with a diameter of 3 mm and the stricture length is kept to be two diameters for all models.

### Boundary conditions

Instead of providing approximate boundary conditions for pulsatile flow simulation (as FFR_CT_), a series of boundary conditions with specified pressures and resistances are applied for steady flow simulation. In the proposed method, a static pressure (88 mmHg) is applied to the inlet. For each outlets, a lumped parameter model with only one resistance is coupled (Fig. 1c). The resistance represents the microvascular resistance. The “form–function” relationship (which relates the resistance of a downstream vessel to the vessel size at each outlet) is assumed to derive the values of microvascular resistance for each outlets with a given total distal microvascular resistance [9, 31]. Finally, a series values of total distal microvascular resistance are specified, providing different outlet boundary conditions for each steady flow simulation. The total distal microvascular resistance is set to be 240 (mmHg s/cm^3^) at first, and then reduced to 87.5, 75.0, 62.5, 50.0, 37.5 and 25.0%. Thus, for one case, steady flow is simulated seven times with seven different total distal microvascular resistances. Since only one outlet exists in each idealized models, the outlet may not be coupled to the lumped parameter model to enforce the “form–function” relationship. Thus, a more simple boundary conditions can be applied to get the pressure-flow curves. For idealized models, a relative zero pressure is assumed at the outlet and a series of static pressure is applied at the inlet. The inlet static pressure is set to be 0.5 mmHg at first, and then increased by 0.5 mmHg each time, until it reaches 11 mmHg.

### Computational method

ANSYS FLUENT V14 (ANSYS Inc.) was used to perform flow simulations. The blood was modeled as an incompressible Newtonian fluid. The dynamic viscosity is set to be 3.5 cP and the density to be 1050 kg/m^3^. The k–ω shear stress transport turbulence model was adopted for turbulence modeling of the low Reynolds number flow in stenosis arteries [32]. Mesh independence was judged by comparing both the computed velocities and the pressure. For each case, further grid refinement (doubled mesh resolution) led to < 1% relative error in velocity and pressure profiles.

### Post-processing

The start and end of a lesion in curved multiplanar reformats are defined by an experienced observer. Then, for each case, the mean pressures at these two planars and the mean flow rate across the stenosis are extracted from each steady flow simulation results. The pressure drop is obtained by subtracting the mean pressure at the end of a lesion from that at the start of a lesion. Finally, *f* and *s* in Eq. 1 are estimated from the Pressure-Flow data by using iterative least squares estimation for nonlinear regression [33]. Two additional parameters are further extracted based on the estimated *f* and *s*. The area (*S*) below the pressure-flow curve (area between flow rate of 0 ml/s to *q* ml/s) can be obtained from the following equation:

2 $$S = \int\limits_{0}^{q} {\left( {f\overline{Q} + s\overline{Q}^{2} } \right)} d\overline{Q} = \frac{f}{2}\left. {\overline{Q}^{2} } \right|_{q} + \frac{s}{3}\left. {\overline{Q}^{3} } \right|_{q}$$

with setting *q* to be 1 and 2 ml/s, respectively, two areas, S_1_ and S_2_, can be calculated from Eq. 2.

### Validation with FFR

To further validate the proposed method, six patients who undergo both FFR and cCTA scans are employed. FFRs are measured with pressure wire (St. Jude Medical, Inc.) by experienced invasive cardiologists. cCTA images are acquired and six 3D coronary models with stenosis in LAD are reconstructed as described in “Vascular Geometry Reconstruction”. Parameters derived from the simulated pressure-flow curve are compared with the invasive FFR.

## Results

**Fig. 2:**
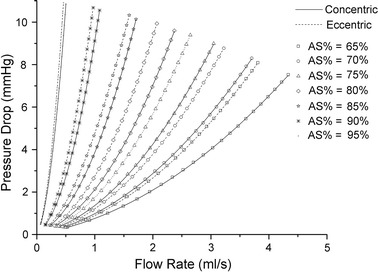
Pressure-flow relationships for idealized models (simulated results and model-predicted results). *Concentric* concentric shaped stenosis models; *Eccentric* eccentric shaped stenosis models; *AS%*: area stenosis

The pressure-flow relationships for idealized models are shown in Fig. 2. As the flow rate increases, the pressure drops across the stenosis section increases for both concentric and eccentric models, and a larger AS% will lead to a more rapid increase in pressure drop. Compared with concentric models, the eccentric models with a same AS% always have a larger pressure drop at given flow rate, and this phenomena will be more pronounced in models with smaller AS %. For each idealized models, the *f* and *s* in Eq. 1 are estimated from the simulated pressure drops and flow rates. From the results, the model-predicted pressure drops (by using Eq. 1 with estimated *f* and *s*) are consistent well with the simulated ones.

**Fig. 3:**
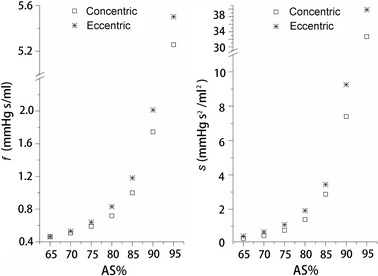
Estimated parameters (*f* and *s*) for idealized models. *Concentric* concentric shaped stenosis models; *Eccentric* eccentric shaped stenosis models; *AS%* area stenosis

The estimated parameters (*f* and *s*) for idealized models with different AS% are shown in Fig. 3. Both *f* and *s* will increase as AS% increases, and a more rapid increase is observed as AS% become greater than 80%. Besides the degree of the stenosis, the shapes also have great impact on the values of the estimated parameters. For models with the same AS%, the estimated *f* and *s* for eccentric stenosis are always greater than that for concentric stenosis.

**Fig. 4:**
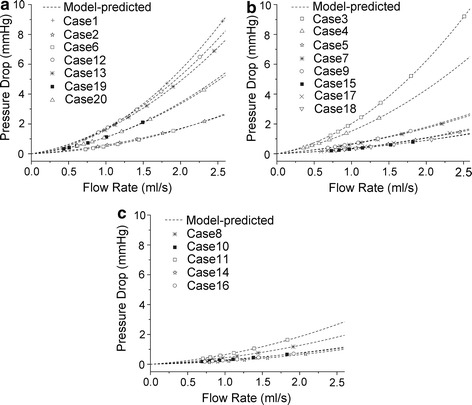
Pressure-flow relationships for reconstructed patient-specific models. **a** Severe group (AS% > 70%); **b** middle group (50% < AS% < 69%); **c** mild group (AS% < 49%). Model-predicted: model-predicted pressure drops

The pressure-flow relationships for reconstructed patient-specific models are shown in Fig. 4. The 20 models are separated into three groups based on AS%: AS% > 70% (severe), 50% < AS% < 69% (middle) and AS% < 49% (mild). However, no obvious differences in pressure-flow relationships are observed between severe stenosis and middle stenosis. Flat pressure-flow curves are observed in two cases in severe group, while sharp pressure-flow curves are observed in two cases in middle group. Five cases in middle group have a similar pressure-flow curves as those in mild group. For all cases, the model-predicted pressure drops (using seven steady flow simulation results) are consistent well with the simulated ones.

**Fig. 5:**
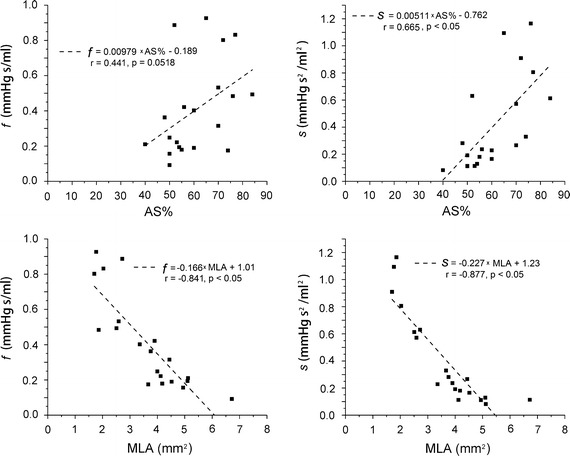
Linear correlation of the estimated parameters (*f* and *s*) with AS% and MLA. *AS%* area stenosis; *MLA* minimal lumen area

Figure 5 shows the linear correlation of the estimated parameters (*f* and *s*) with AS% and MLA. When correlated with AS%, only *s* show a moderate but significant correlation (r = 0.66, P < 0.05). However, when correlated with MLA, both *f* and *s* have linear and significant correlations (*f*: r = 0.84, P < 0.05; *s*: r = 0.88, P < 0.05).

**Fig. 6:**
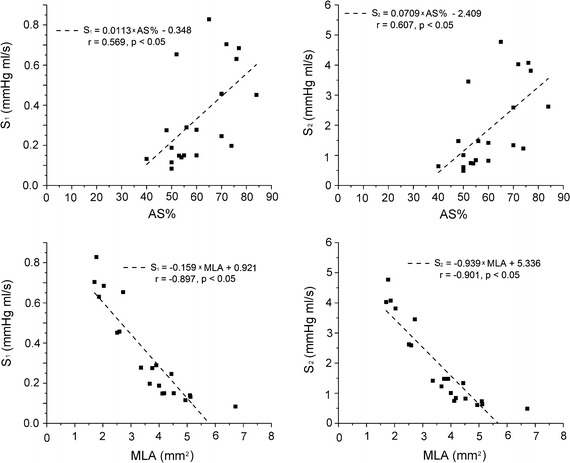
Linear correlation of the extracted parameters (S_1_ and S_2_) with AS% and MLA. *AS%*: area stenosis; *MLA* minimal lumen area

Figure 6 shows the linear correlation of the extracted parameters (S_1_ and S_2_) with AS% and MLA. For S_1_, it has a moderate but significant correlation with AS% (r = 0.56, P < 0.05) and a linear and significant correlation with MLA (r = 0.90, P < 0.05). Similar results are observed for S_2_ (correlated with AS%: r = 0.61, P < 0.05; correlated with MLA: r = 0.90, P < 0.05).

**Fig. 7:**
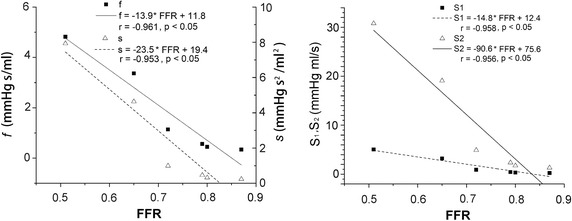
Linear correlation of the four parameters (*f*, *s*, S_1_ and S_2_) with invasive FFR

Figure 7 shows the linear correlation of the estimated parameters with invasive FFR. All the four parameters show a linear and significant correlations with invasive FFR (*f*: r = 0.96, P < 0.05; *s*: r = 0.95, P < 0.05; *S*_1_: r = 0.96, P < 0.05; *S*_2_: r = 0.96, P < 0.05).

## Discussion

A promising noninvasive method was proposed for assessing of hemodynamic significance of coronary stenosis. Anatomy information of a stenosis is first derived from the cCTA images; and then, the pressure-flow relationship of a stenosis is obtained by using CFD method. Unlike previous invasive (FFR and CDP) or noninvasive method (FFR_CT_) which evaluating the stenosis at a specific flow rate (maximum hyperemia), the proposed method characters the hemodynamic stenosis at a series of flow rate. The main advantages of the proposed method are: (1) it is a non-invasive functional diagnosis method; (2) do not need to model the maximum hyperemia condition; (3) fully characters the pressure-flow behavior of a stenosis within normal physiological range. The proposed method is validate in six patients who undergo both FFR and cCTA scans, and all the proposed four parameters have linear and significant correlations with invasive FFR.

**Fig. 8:**
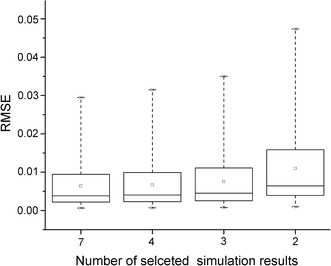
RMSEs of the predicted pressure drops for models fitted with different selections of the steady flow simulation results. 7: all 7 steady flow simulation results are selected for estimation; 4: 4 steady flow simulation results (with total distal microvascular resistance set to be 100, 75, 50 and 25% of the rest level) are selected for estimation; 3: 3 steady flow simulation results (with total distal microvascular resistance set to be 100, 62.5 and 25% of the rest level) are selected for estimation; 2: only 2 steady flow simulation results (with total distal microvascular resistance set to be 100% and 25% of the rest level) are selected for estimation

CFD is always combined with cardiovascular imaging for blood flow analysis in various cardiac disease [34–39]. However, it is usually quite time-consuming for clinical use due to the complex computational models. To overcome this problem, steady flow simulation is used in our proposed method. And the two parameters, *f* and *s*, which are estimated with seven steady flow simulation results, can be estimated even with only two steady flow simulation results. The root-mean-square errors (RMSE) of the predicted pressure drops for models fitted with different numbers of selected steady flow simulation results are shown in Fig. 8. It is demonstrated that the pressure-flow curves can be fitted well with much less computation time (only needs two steady flow simulation results).

### Compared with cCTA

It was reported that anatomic assessments of stenosis evaluated by cCTA had significant correlation with those evaluated by digital subtraction angiography [40]. Although cCTA alone can accurately detect anatomically obstructive coronary stenosis, it cannot define the hemodynamic significance of a stenosis due to the complex relationship between stenosis and flow [1]. The spatial characteristics of stenosis have great impact on flow [41–43]. Even for stenosis with similar AS%, the pressure-flow behaviors still can be different due to the different shapes of stenosis (as shown in Figs. 2 and 4). With the anatomic models obtained by cCTA, a further CFD analysis is performed to fill the gap between anatomically obstructive and hemodynamic significance. In the proposed method, pressure-flow curves are obtained and further employed to define the hemodynamic significance. It characters the nonlinear relationship between the flow rate and the pressure drop across a stenosis, reflecting the flow resistances of a given stenosis. A sharper curve represents a larger flow resistance, while more flat curve means smaller flow resistance. As shown in Fig. 4, severe stenosis defined by cCTA can have a relative smaller flow resistance, but it cannot be known before the pressure-flow curve obtained. Several parameters are derived from the curves to quantify the flow resistance. As compared with AS%, flow resistance has a more direct relationship with the radius of a stenosis [44]. Thus, from the results, moderate correlations are observed when correlated these parameters with AS%, but linear and significant correlations are obtained when correlated with MLA.

### Compared with FFR_CT_

CFD is applied in both FFR_CT_ and proposed method to provide the hemodynamic information of a stenosis. For FFR_CT_, pressure drop during maximal hyperemia is employed [9]; while for our method, pressure-flow relationship is derived. With a given flow rate, the pressure drop across the stenosis can be determined from its pressure-flow relationship. Thus, FFR_CT_ can be derived from the obtained pressure-flow relationship if the exact flow rate during maximal hyperemia is known. However, the main problem for CFD based method is that there is no additional measurement about the flow rate during maximal hyperemia [17]. Although clinical trials (DISCOVER-FLOW, DeFACTO and NXT) demonstrated that FFR_CT_ improved diagnostic accuracy in differentiating ischemic stenosis as compared with cCTA alone [19–21], the performance is still unsatisfactory when compared with invasive FFR. One possible reason is that FFR_CT_ has to employ several physiological models to estimate an approximate maximal hyperemia condition. Instead of modeling maximal hyperemia condition, a series of boundary conditions are specified and those simulated results are combined to provide a pressure-flow curve in proposed method. By this way, the accuracy do not rely on the modeling of maximal hyperemia condition. Additionally, pressure-flow relationship represents the flow resistances of a stenosis, which is determined mainly by its anatomical morphology. A functionally significant stenosis will increase the flow resistances and lead to a decrease in distal perfusion pressure, further cause ischemia [23, 43]. Thus, flow resistance maybe a more essential index for functional measurement of stenosis, as compared with pressure derived index.

### Compared with CDP

CDP is a parameter which incorporates both pressure and flow variations [26]. By ignoring the viscous friction effect and replacing the flow rate as flow velocity in Eq. (1), the expansion loss (*s*) can be approximately obtained with a single measurement of pressure drop and flow velocity. In the presence of microvascular dysfunction and submaximal hyperemia, both the pressure drop and the flow velocity will decreased, leading to a significant increase of pressure-derived parameters (FFR and FFR_CT_), but it has a limited impact on CDP [27]. Ideally, the flow rate should have little impact on CDP. However, due to the assumptions made by CDP, the measured values in basal and hyperemic conditions are still quite different [30]. Additionally, it is also difficult to measure the flow velocity accurately. Fortunately, those problem can be overcome by the proposed CFD based method. By combining several measurements, both the viscous friction (*f*) and expansion loss (*s*) can be estimated and the pressure-flow relationship can be obtained. With the CFD method, the flow rate also can be measured without the impact of guide wire insertion. It is reported that CDP has the ability to localize the differential culpability for flow impediment: translesional or microvascular [27–29]. But in proposed method, it is noninvasive and only the anatomy information derived from cCTA is used, thus the microvascular disease is excluded and it is concentrated to evaluate the culpability for flow impediment of translesional stenosis.

## Limitations

In this paper, a CFD based non-invasive method is proposed for functional measurement of coronary stenosis, which do not need to model the maximal hyperemia condition. However, only six patients are employed to validate with current “gold standard” invasive FFR. Besides, due to lack of FFR data, currently there is no cut-off value for those parameters derived from pressure-flow curves. Even though, this is a first approach to functional measurement of coronary stenosis non-invasively without modeling the maximal hyperemia conditions. Given this advantage over FFR_CT_, we believe that it will have a promising role in clinical practice. Still, further validations need to be performed, especially corrected with FFR. To limit the computational time, steady state flow is simulated to get the pressure-flow curves. However, blood flow is pulsating, which is inconsistence with steady state flow assumption. Fortunately, current “gold standard” invasive FFR is based on time-averaged pressure measured over several cardiac cycles [4], and previous studies also demonstrated that FFR_CT_ obtained by using steady state flow simulation still performed well [45]. Additionally, the boundary conditions used for pulsating flow simulation in FFR_CT_ is derived from lumped parameter models, which is also inconsistence with real conditions [11, 46]. Thus, steady state flow is simulated in our method.
